# Unravelling the gut-lung axis: insights into microbiome interactions and Traditional Indian Medicine's perspective on optimal health

**DOI:** 10.1093/femsec/fiad103

**Published:** 2023-09-01

**Authors:** Swati Haldar, Snehal R Jadhav, Vandana Gulati, David J Beale, Acharya Balkrishna, Anurag Varshney, Enzo A Palombo, Avinash V Karpe, Rohan M Shah

**Affiliations:** Drug Discovery and Development Division, Patanjali Research Institute, NH-58, Haridwar 249405, Uttarakhand, India; Consumer-Analytical-Safety-Sensory (CASS) Food Research Centre, School of Exercise and Nutrition Sciences, Deakin University, Burwood, VIC 3125, Australia; Biomedical Science, School of Science and Technology Faculty of Science, Agriculture, Business and Law, University of New England, Armidale, NSW 2351, Australia; Environment, Commonwealth Scientific and Industrial Research Organisation (CSIRO), Ecosciences Precinct, Dutton Park, QLD 4102, Australia; Drug Discovery and Development Division, Patanjali Research Institute, NH-58, Haridwar 249405, Uttarakhand, India; Department of Allied and Applied Sciences, University of Patanjali, Patanjali Yog Peeth, Roorkee-Haridwar Road, Haridwar 249405, Uttarakhand, India; Drug Discovery and Development Division, Patanjali Research Institute, NH-58, Haridwar 249405, Uttarakhand, India; Department of Allied and Applied Sciences, University of Patanjali, Patanjali Yog Peeth, Roorkee-Haridwar Road, Haridwar 249405, Uttarakhand, India; Department of Chemistry and Biotechnology, School of Science, Computing and Engineering Technologies, Swinburne University of Technology, Hawthorn, VIC 3122, Australia; Department of Chemistry and Biotechnology, School of Science, Computing and Engineering Technologies, Swinburne University of Technology, Hawthorn, VIC 3122, Australia; Socio-Eternal Thinking for Unity (SETU), Melbourne, VIC 3805, Australia; Agriculture and Food, Commonwealth Scientific and Industrial Research Organisation (CSIRO), Acton, ACT 2601, Australia; Department of Chemistry and Biotechnology, School of Science, Computing and Engineering Technologies, Swinburne University of Technology, Hawthorn, VIC 3122, Australia; School of Health and Biomedical Sciences, STEM College, RMIT University, Bundoora West, VIC 3083, Australia

**Keywords:** COVID-19, dysbiosis, Gut-lung axis, microbiome, traditional medicine

## Abstract

The microbiome of the human gut is a complex assemblage of microorganisms that are in a symbiotic relationship with one another and profoundly influence every aspect of human health. According to converging evidence, the human gut is a nodal point for the physiological performance matrixes of the vital organs on several axes (i.e. gut-brain, gut-lung, etc). As a result of COVID-19, the importance of gut-lung dysbiosis (balance or imbalance) has been realised. In view of this, it is of utmost importance to develop a comprehensive understanding of the microbiome, as well as its dysbiosis. In this review, we provide an overview of the gut-lung axial microbiome and its importance in maintaining optimal health. Human populations have successfully adapted to geophysical conditions through traditional dietary practices from around the world. In this context, a section has been devoted to the traditional Indian system of medicine and its theories and practices regarding the maintenance of optimally customized gut health.

## Teaser

The role of traditional Indian system of medicine in managing the dysbiotic microbiome in the gut-lung axis is discussed in this review.

## Introduction

The human body is inhabited by a community of microorganisms that live in a symbiotic relationship with their host. Microbiota is the term used to describe the collection of microorganisms, including bacteria, archaea, and some unicellular eukaryotes, that inhabit a specific environment or niche, such as the gut, mouth, or skin, etc. (Lederberg and McCray 2001, Marchesi and Ravel 2015). The microbiome includes the community of microorganisms (*i.e*. microbiota) and the molecules that they produce (Whipps et al. 1988, Berg et al. 2020). Among these molecules are nucleic acids, proteins, carbohydrates, and lipids, as well as their metabolites, including toxins, organic and inorganic molecules, signaling molecules, and molecules produced by coexisting hosts and influenced by the environment around them. The microbiome also includes other genetic elements such as viruses and plasmids, which are capable of moving from one organism to another. They form a complex ‘*theatre of activity*’ that influences many aspects of human physiology and health (Berg et al. 2020).

Humans are now considered to have two genomes: an inherited genome from a person's biological parents, and the acquired genome post-birth (*i.e*. the microbiome) (Grice and Segre 2012). The inherited genome remains relatively stable throughout a person's lifetime, whereas the acquired microbiome is dynamic and strongly shaped by factors such as age (Zapata and Quagliarello 2015), diet (David et al. 2014), lifestyle (Song et al. 2020), travel (Voorhies and Lorenzi 2016), geography (Yatsunenko et al. 2012), hormonal cycles (Koren et al. 2012), illness (Cho and Blaser 2012, Pflughoeft and Versalovic 2012) and drug interventions/therapies (Tapiainen et al. 2019, Ribeiro et al. 2020). The human microbiome plays a pivotal role in homeostatic regulation, the development of the immune system, food digestion, and detoxification (D'Argenio and Salvatore 2015).

In this current review, we provide an overview of the ecology and the functional role of the gut and airway microbiome. The communication between the gut and lung (‘*gut-lung axis*’) provides mechanistic insights into how the (‘*dysbiotic*’) gut microbiota may affect respiratory immunology and lung health (e.g. COVID-19 infections) is also discussed here. We further discuss the role of probiotics and prebiotics on the microbiome and the prevention and treatment approaches of the traditional Indian system of medicine.

## Microbial ecology and function in the healthy human gut and respiratory tract

**Figure 1.**
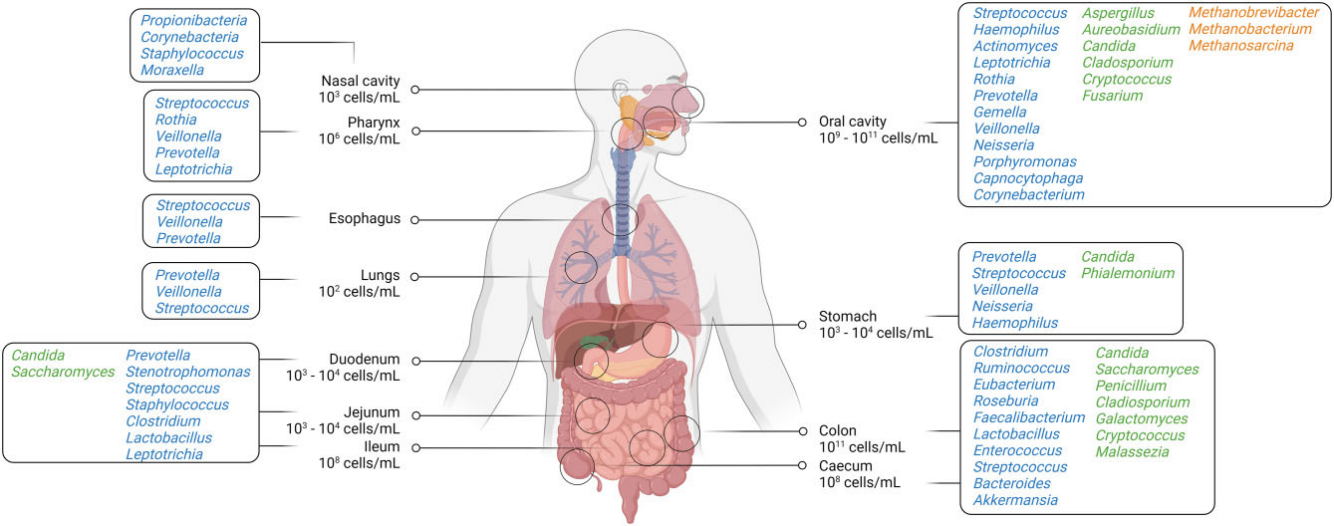
Microbial composition among the physiological niches of the human respiratory and gastrointestinal tracts. The most abundant bacteria within the niches are denoted in blue text, the most abundant fungi in green, and the most abundant archaea in orange. *Please note, the numbers presented in this figure represent the abundance of bacterial members in the human gastrointestinal and respiratory tracts. The focus of this figure is primarily on bacteria due to the limited knowledge available regarding the specific abundance of fungi and archaea in the human gastrointestinal tract*. Created with BioRender.com.

Microbial ecology, the study of the interactions between microorganisms and their environment, plays a crucial role in understanding the structure and function of microbial communities in the human gut and respiratory tract. The persistence of microbes in the gut is influenced by the complex interplay of factors, including differences in physiological niches and downstream microbial colonization (de Vos et al. 2022). The gut environment differs markedly between anatomical regions in terms of physiology, flow rates, substrate availability, pH, oxygen concentrations, redox potential, host secretions (i.e. mucus, bile, and antibodies), the gut architecture, peristalsis, and transit times (de Vos et al. 2022). Furthermore, the microbiota complexity varies, and bacterial load increases gradually along the gastrointestinal tract (Shah et al. 2020). While fungi and archaea also contribute to the gut microbiota, their load in the gastrointestinal tract are still not well-characterized. Fig. 1 illustrates the microbial composition, and the typical order of magnitude estimates for the number of bacteria that inhabit different physiological niches of the human gastrointestinal and respiratory tract (Pei et al. 2004, Chen et al. 2010, Ghannoum et al. 2010, Matarazzo et al. 2011, Blaut 2018, Rajilic-Stojanovic et al. 2020). The gut microbiome provides the human host with several essential functions. The gut microbiota converts indigestible foods into metabolites that are easily absorbed (Tremaroli and Bäckhed 2012), synthesizes essential vitamins (LeBlanc et al. 2013), removes toxic compounds (Claus et al. 2016), outcompetes pathogens (Sommer and Bäckhed 2013), strengthens the intestinal barrier (Abreu 2010), and stimulates and regulates the immune system (Abreu 2010, Hooper Lora et al. 2012). Most of these functions are interconnected and tightly intertwined with human physiology.

Similarly, the respiratory tract microbiome is shaped by environmental exposures and physiological parameters such as pH, temperature, and oxygen and carbon dioxide levels. Our current understanding of the airway microbiome has been relatively limited compared to the gut microbiome, which has been the focus of most microbiome studies, especially in clinical settings. Nonetheless, the field of airway microbiome research has shown significant progress in recent years. Although less abundant compared to the gut microbiome, numerous studies have shown that the airway microbiome is a significant component of the airway ecosystem that is also connected with the immune system (Adami and Cervantes 2015, Lee et al. 2016, Freeman and Curtis 2017, Huffnagle et al. 2017, Stavropoulou et al. 2021, Yagi et al. 2021). Microbial spread in the airways is considerably different from that of the gut region. The gradient is a result of a greater aerobic environment, variable temperature (due to gas exchange), and coating of bacteriostatic lipopolysaccharide across the alveolar surface (Huffnagle et al. 2017). In humans, the airway microbiome differs from the gut microbiome in its developmental timeline, with the former largely established between birth and three years of age. This process is mainly shaped by environmental factors, rather than the transmission of microbes from the mother to the child during birth (Fragkou et al. 2021).

## The gut-lung axis

**Figure 2.**
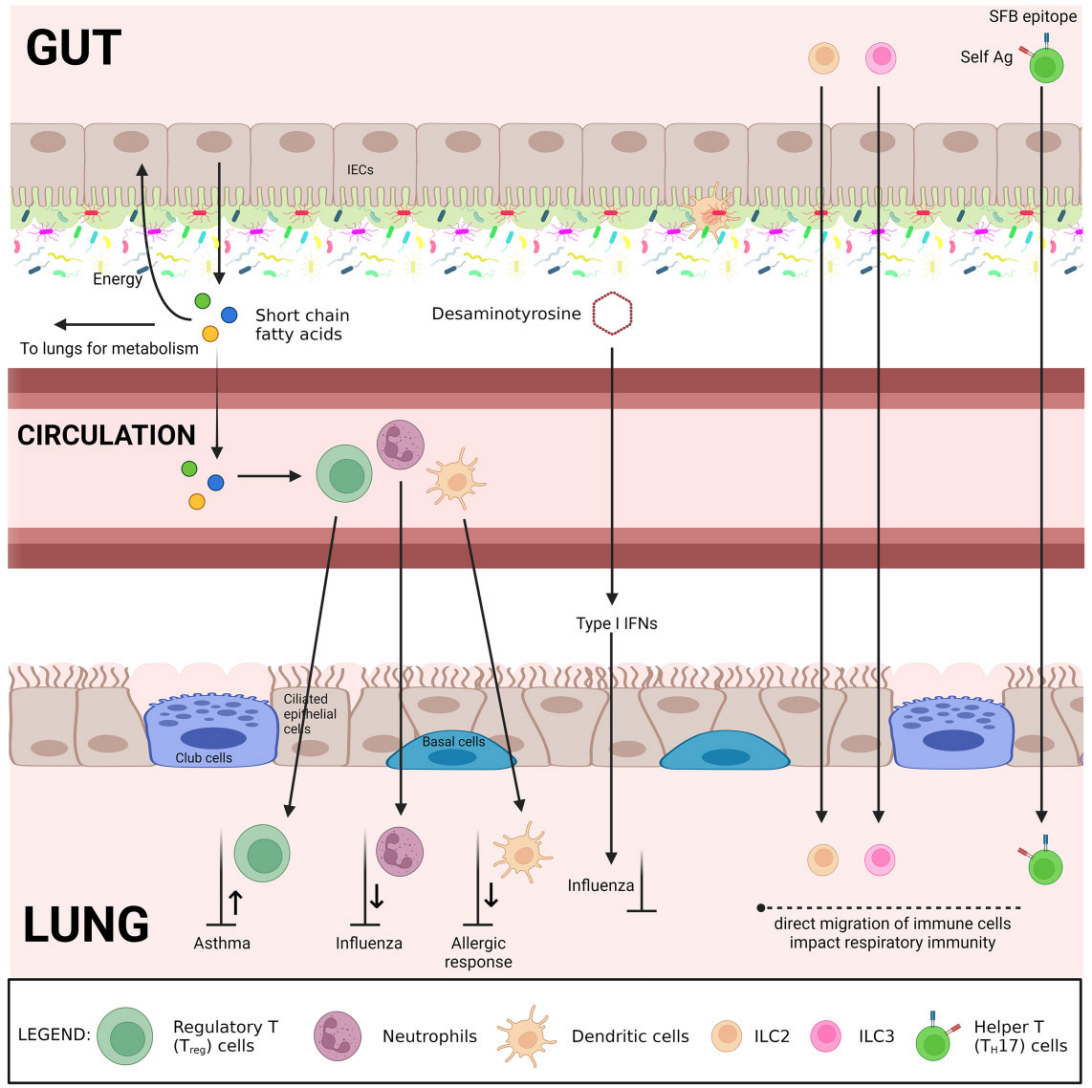
Schematic representation illustrating the concept of cross-talk between the gut and lung in the context of the human gut-lung axis. SFB: segmented filamentous bacteria, Ag: antigen, ILC: innate lymphoid cells, IFN: interferon, IEC: intestinal epithelial cells. *Please note, this figure is not intended to provide an anatomically accurate depiction of the organs or their precise spatial arrangement. Rather, it serves to visually demonstrate the communication and interactions between these systems. The figure highlights the interplay between the gut and lung and their potential influence on each other's microbiomes. Please note that the spatial relationships and anatomical details are simplified for conceptual clarity. This figure is intended to support the discussion on the role of traditional medicines in managing the gut-lung axis, rather than providing detailed anatomical information*. Created with BioRender.com.

As distant as they are physical, the respiratory and gastrointestinal tracts share the same embryonic origin and common structure, suggesting that their interactions may be multifaceted. There is a clear crosstalk between the two sites known as the gut-lung axis, which was reported recently (Budden et al. 2017). The gut–lung axis refers to the reciprocal exchanges of microorganisms and/or their metabolites and immunomodulatory signals between the gut and lungs (Fig. 2). While such crosstalk occurs in both directions, the mechanisms underlying these transfers are not well understood. However, there is considerable evidence in the literature that indicates the role of microbial crosstalk in respiratory disorders.

### Microbial crosstalk between the gut and lungs

The mucosal epithelial surfaces of the gastrointestinal and respiratory tracts are continuously exposed to a variety of microorganisms. The microorganisms can gain access due to inhalation or ingestion, and the transfer of microorganisms from the oral cavity and upper gastrointestinal tract to the lung can occur due to aspiration (Budden et al. 2017). While the gut and the respiratory tract microbiota are very similar at the phylum structure level, the members that dominate these phyla are very different. Actinobacteria, Firmicutes, and Bacteroidetes predominate in the gut, and Proteobacteria, Firmicutes and Bacteroidetes dominate in the lungs (Trivedi and Barve 2020). The gut and lung microbiota show a close relationship throughout life, indicating a host-wide network between them (Grier et al. 2018). It was also reported that gut and lung abundances are highly correlated over time (Madan et al. 2012). Madan et al. (2012) suggested that changes in diet alter gut colonisation patterns and that colonization with *Roseburia, Dorea, Coprococcus, Blautia*, or *Escherichia* presaged their appearance in the respiratory tract. The literature on the direct transfer of microorganisms between the gut and lungs is very limited. Various hypotheses have been postulated to explain gut-lung translocation such as the gut-lymph theory (Trivedi and Barve 2020) or the common mucosal immune system (He et al. 2020). Scientific evidence shows that the barrier integrity is compromised in cystic fibrosis, sepsis, and acute respiratory distress syndrome suggesting gut-lung translocation of microorganisms (Madan et al. 2012, Dickson et al. 2016). Similarly, Dickson et al. (2016) demonstrated that the lung microbiota is enriched with gut bacteria, such as *Bacteroides* spp. after sepsis and acute respiratory distress syndrome.

### Perturbation of the gut-lung axis by bacterial components and metabolites

The gut microbiota communicates with the lungs via soluble microbial components and bacterial metabolites which reach the systemic circulation. Microbial components include pathogen-associated molecular patterns (PAMPs), such as peptidoglycans and lipopolysaccharides. Host cells that express pattern recognition receptors such as Toll-like receptors (TLRs) or nucleotide-binding and oligomerization domain (NOD)-like receptors (NLRs) can recognise peptidoglycans and lipopolysaccharides, among other PAMPs (Wypych et al. 2019). The TLRs are a well-characterised family of receptors, widely expressed in immune cells (including macrophages, mast cells, natural killer cells, neutrophils, eosinophils, basophils, and dendritic cells) and body cells (including intestinal epithelial cells). The activation of TLRs induces the activation of antigen-presenting cells and triggers the signalling cascade to encounter the invading microbes and/or repair the damaged tissue. Excessive activation of TLRs disrupts immune homeostasis and leads to sustained production of pro-inflammatory mediators, thereby increasing the risk of inflammatory diseases and autoimmune disorders (de Vos et al. 2022).

The host also senses microbial metabolites (e.g. short-chain fatty acids; SCFAs) produced from the metabolism of indigestible nutrients (e.g. dietary fibres). Most of the SCFAs are either consumed by the colonocytes for energy or used by the epithelial cells in the gut to shape local immunity (Fig. 2). The remainder is transported to the liver via the portal vein for metabolism. Any unmetabolised SCFAs are then redistributed through the circulation to peripheral tissues (Yip et al. 2021). For example, in bone marrow, SCFAs influence immune cell development (Wypych et al. 2019) by impairing the ability of dendritic cells (DCs) resulting in an attenuated allergic response (Trompette et al. 2014, Cait et al. 2018), or by promoting differentiation of regulatory T cells (T_reg_ cells) resulting in a reduced asthmatic response (Thorburn et al. 2015), or by reducing neutrophil recruitment to the airways during influenza (Trompette et al. 2018) (Fig. 2). Desaminotyrosine, produced by an obligate clostridial anaerobe from the digestion of plant flavonoids, was found to protect from influenza through type I interferons (Steed et al. 2017) (Fig. 2).

### Migration of immune cells

Another significant route of communication in the gut-lung axis involves the migration of immune cells from the intestine to the respiratory tract through circulation. Pu et al. (2021) demonstrated that the gut microbiota directs the migration of innate lymphoid cells type 2 (ILC2) from the gut to the lung via the gut–lung axis. ILC2s play a critical role in regulating the immune response, controlling bacterial infections, and maintaining lung homeostasis. The host produces interleukin (IL)-33 in response to an increase in Proteobacteria within the gut microbiome, which plays a critical role in facilitating the natural migration of ILC2 (Pu et al. 2021) (Fig. 2). The sensing of commensal bacteria by intestinal DCs induces the secretion of IL-22. These cytokines are needed to prime the mucosal ILC3 to migrate from the intestine to the lungs (Gray et al. 2017) (Fig. 2). However, these cells have different roles in the immune system, with ILC3s being involved in the maintenance of mucosal immunity in the gut and respiratory tract, while ILC2s primarily regulate allergic and anti-parasitic responses. ILC3s produce cytokines such as IL-22 and IL-17 to protect against infections and maintain the integrity of the mucosal barrier, while ILC2s produce cytokines such as IL-5 and IL-13 to activate eosinophils and promote IgE antibody production. Furthermore, this crosstalk between gut and lung might also occur during diseases, e.g. the migration of T_H_-17 cells co-expressing T cell antigen receptors that recognize segmented filamentous bacteria (SFB) epitope and self-antigen to lungs, contributing to lung pathology (Bradley et al. 2017) (Fig. 2). Another example of the gut-lung axis crosstalk can be seen in the case of fungal overgrowth in the gut. *Candida* spp. (as well as many other fungi) directly produce prostaglandin E2 (PGE_2_), a potent immunomodulator that is produced by immune cells from host arachidonic acid (Underhill and Iliev 2014). Kim et al. (2014) demonstrated that PGE_2_ produced due to the overgrowth of commensal *Candida* species in the gut can reach the lungs through the bloodstream, induce macrophage polarisation in the lungs, and influence systemic responses, including allergic inflammation.

## Dysbiosis: detrimental deviation in microbiome

**Figure 3.**
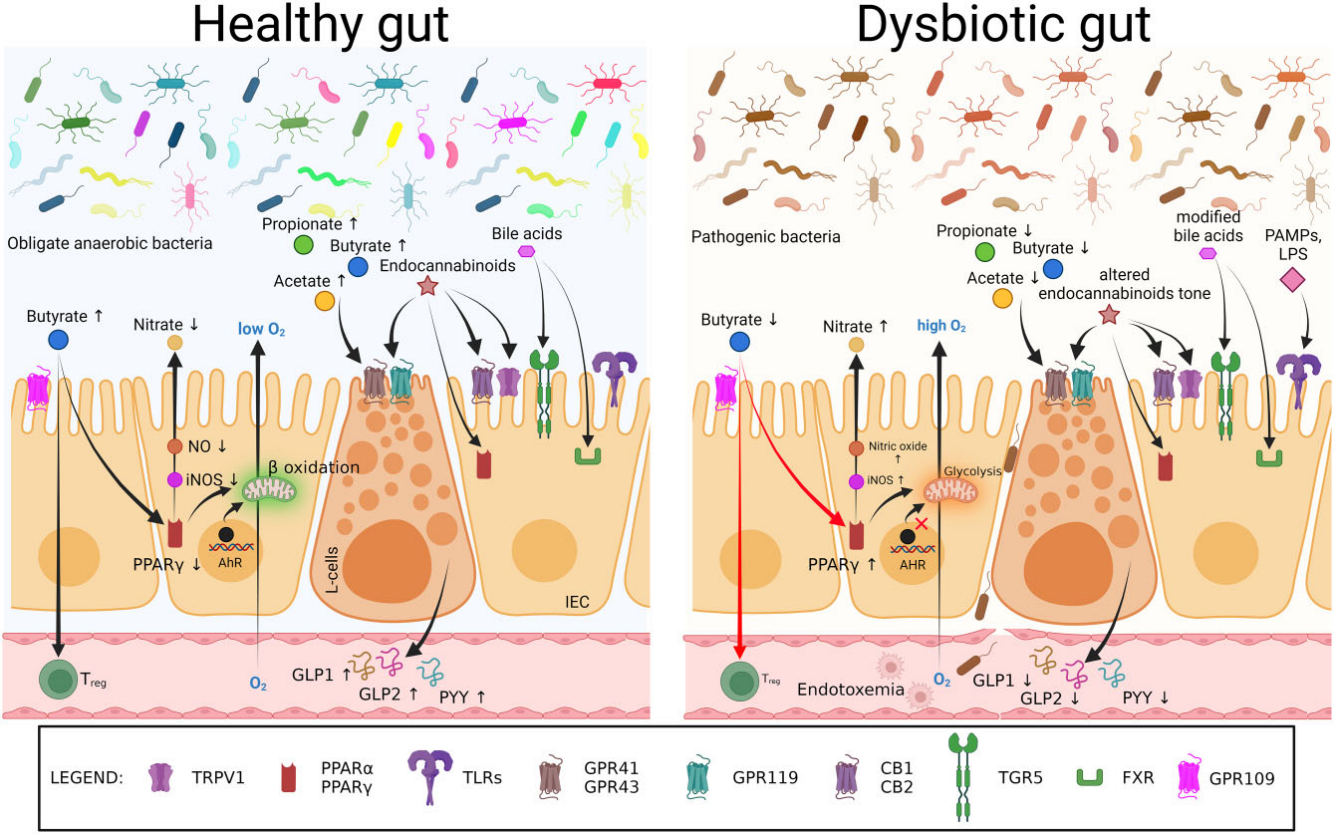
Molecular links between gut microbiota and host health in healthy and dysbiotic state. In a healthy gut, intestinal epithelial cells (IECs) utilise butyrate through mitochondrial beta-oxidation, maintaining an anaerobic environment. Butyrate binds to peroxisome proliferator-activated receptor gamma (PPARγ), reducing inducible nitric oxide synthase (iNOS) expression and nitric oxide (NO) production. G-protein-coupled receptor (GPR)109 serves as a major receptor for butyrate. In dysbiosis, low butyrate levels decrease PPARγ activity, increase glycolysis, and elevate iNOS expression, leading to an increased NO production and nitrates. Butyrate stimulates immune cells, like regulatory T cells (T_reg_), through GPR109, exerting anti-inflammatory effects. Reduced aryl hydrocarbon receptor (AhR) activity disrupts gut barrier function. short-chain fatty acids (SCFAs: butyrate, propionate and acetate), endocannabinoids, and bile acids activate receptors on L-cells and IECs, promoting gut peptide secretion. SCFAs activate GPR41 and GPR43 on L-cells, promoting secretion of gut peptides including glucagon-like peptide-1 (GLP-1), GLP-2, and peptide YY (PYY). Endocannabinoids interact with cannabinoid receptors type 1 (CB1), CB2, PPARα, PPARγ, and transient receptor potential vanilloid type-1 (TRPV1) receptors, while bile acids activate farnesoid X receptor (FXR) and Takeda G protein-coupled receptor 5 (TGR5) receptors. Gut pattern recognition receptors such as toll-like receptors (TLRs) detect pathogen-associated molecular patterns (PAMPs) and lipopolysaccharides (LPS) from the microbiota. These interactions reduce intestinal permeability, enhance insulin sensitivity and secretion, decrease food intake, lower plasma lipids, and mitigate hepatic steatosis and endotoxemia risk, associated with reduced inflammation. Dysbiosis leads to opposite effects. Overall, interactions among gut microbiota, IECs, immune cells, and molecular receptors maintain gut homeostasis and impact metabolism and inflammation. Created with BioRender.com.

**Figure 4.**
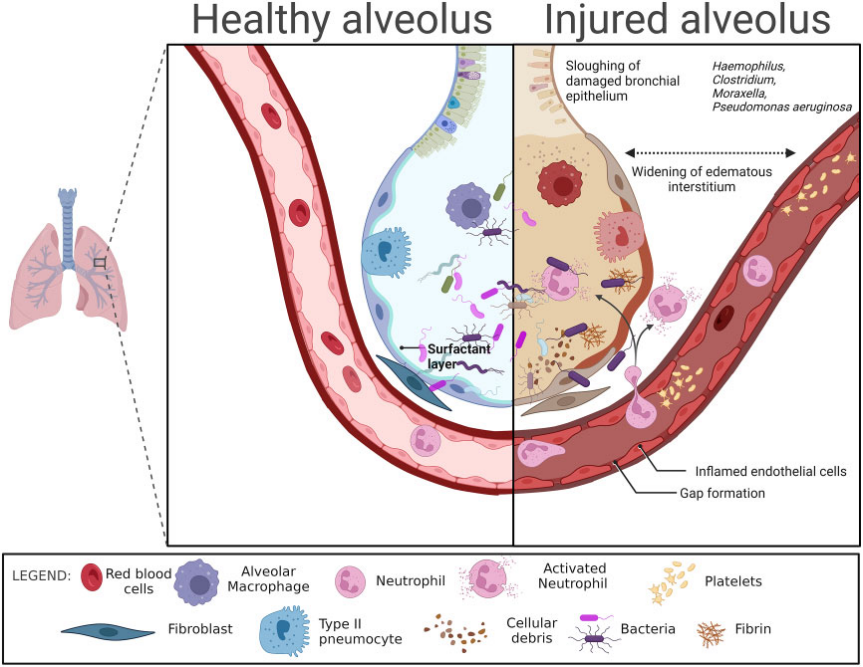
The microbiome-immune proteins-cellular interaction in the lungs. Created with BioRender.com.

The microbiome plays an important role in maintaining homeostasis in a healthy body. Many of the modern multifactorial diseases such as obesity, allergies, diabetes, asthma, inflammatory bowel disease (IBD), and neurodegeneration that are becoming more common have been linked to ‘*dysbiosis*’, an aberrant microbiome structure that alters the taxonomic composition as well as the metagenomic function of the microbial community with the human host. Dysbiosis is defined as a change in the composition and function of the microbiota, caused by a combination of environmental and host-related factors that disrupt the commensal ecosystem to a degree that exceeds its resistance and resilience capabilities (Figs 3 and 4).

Dysbiosis continues as a stable condition once the microbiota configuration is modified, and it can take on numerous compositional expressions depending on the causative influence (David et al. 2014). The commensal microbial community in the human body can be thought of as a stable system, where healthy and dysbiotic states exist in different configurations, akin to an energy landscape. However, for the system to transition from one state to another, external forces stronger than the system's inherent stability are required. In simpler terms, the human microbial community can exist in both healthy and unhealthy states, but a significant disruption is necessary to shift the system from one state to another (Lloyd-Price et al. 2016). The enormous variability in taxonomic microbiota composition among healthy individuals due to geographical distribution, age, and dietary habits raises the question of what constitutes a reference population, allowing almost any gut microbial configuration to be classified as ‘*dysbiotic*’ when compared to a specific control. Even *in vivo* experimental models of dysbiosis are not able to address this lacuna. The microbiome's adaptations to altered environmental conditions or changes in the host's state ultimately result in atypical community composition and function and can have favourable, neutral, or detrimental effects on the host. Adaptive changes in the microbiome in response to perturbations of the steady state, like host tissue deviations from homeostasis, may be harmful if the microbial community does not return to its previous state after normalisation of environmental conditions, and instead persists chronically in a ‘maladaptive’ state with adverse repercussions for the host (Fonseca et al. 2015, Thaiss et al. 2016). Therefore, it would be rather appropriate to define dysbiosis as a microbial community state within the human body that is not just statistically related to the disease but also functionally contributes to the disease's aetiology, diagnosis, or treatment.

Dysbiosis is characterized by an explosion in pathobiont population (Frank et al. 2007, Garrett et al. 2007, Stecher et al. 2013), reduction in the commensal population due to microbial death or reduced bacterial multiplication (Korem et al. 2015), and compromised alpha diversity of commensals due to dysbiosis caused by aberrant food composition (Sonnenburg et al. 2016), IBD (Norman et al. 2015), AIDS (Monaco et al. 2016), and type 1 diabetes (T1D) (Kostic et al. 2015), among other conditions (Mosca et al. 2016). The most common causal factors of dysbiosis are infection and inflammation, age, xenobiotics, diet, genetic makeups of both host and microbiota, heredity, mutations, and other postulated miscellaneous factors, like, circadian disruption (Thaiss et al. 2014, Voigt et al. 2016), physical injury (Kigerl et al. 2016), maternal high-fat diet (Buffington et al. 2016), pregnancy (Koren et al. 2012), and lifestyle (Chen et al. 2022).

Experimentally, dysbiosis was first observed in mouse models of *Citrobacter rodentium* (Lupp et al. 2007) and *Salmonella enterica* subsp. enterica serovar Typhimurium infection (Stecher et al. 2007). Inflammation-induced expansion of *Enterobacteriaceae* members can enhance the development of colorectal cancer (Arthur et al. 2012) and sepsis (Ayres et al. 2012), in addition to intestinal infection. The release of nutrients (Ng et al. 2013), the use of metal ions (Deriu et al. 2013), inter-microbial competition and horizontal gene transfer (Stecher et al. 2012), the exploitation of antimicrobial peptides (Behnsen et al. 2014), and the harnessing of aerobic and anaerobic cellular respiration (Winter et al. 2013, Lopez et al. 2016) are all molecular mechanisms that lead to the establishment of *Enterobacteriaceae* in the inflamed gut.

Diet has significant short (David et al. 2014) and long-term (Wu et al. 2011) impacts on the microbiota composition in the intestine. Dietary xenobiotics could modify homeostatic commensal colonization in addition to negatively affecting the nutrition content of the food. This is most obvious in the case of antibiotics (Cho et al. 2012), but it has also been observed for non-caloric artificial sweeteners (Suez et al. 2014) and dietary emulsifiers (Chassaing et al. 2015), though the mechanisms by which the latter two influence the microbiota is unknown. Dysbiosis caused by diet and xenobiotics is an important driver of illness symptoms in mice (Yoshimoto et al. 2013) and, in some situations, humans (Zhu et al. 2016). In addition to the parameters described above, host genetics play a significant role in the composition of the microbiota, particularly, that of the gut (Levy et al. 2015). Genome-wide association studies that linked genetic loci with microbial taxa and functional pathways (Bonder et al. 2016, Turpin et al. 2016) discovered this and other associations. In addition, many human loci involved in immunological and metabolic activities, including the one encoding for the human vitamin D receptor, were identified as potential drivers of microbial regulation through host genetics (Wang et al. 2016). It is important to consider that dysbiosis is not limited to the gut. Epithelial cell mutations were found to lead to lung microbiome dysbiosis in patients diagnosed with squamous cell carcinoma (Greathouse et al. 2018). The epithelial cell mutations were attributed to smoking. Similarly, in the case of lung cancer patients, the enriched *Acidovorax temporans* population was associated with tumour suppressor gene TP53 mutations, which cause epithelial impairment in the lungs.

## Lung microbiome and gut-lung axis: a COVID-19 perspective

Recent research has shown that the composition of upper airway bacteria is altered during COVID-19 infection. While *Veillonella parvula* was observed to be more abundant during moderate infection, *Actinomyces meyeri* and *Halomonas* spp. were more abundant during mild conditions. This differential abundance suggests a possible role for these species in modulating the immune response to COVID-19 infection, which may lead to increased metabolic activity during mild or moderate disease (Devi et al. 2022).

Nardelli et al. (2021) studied both upper and lower respiratory regions during COVID-19. They observed that the abundance of *Veillonella, Staphylococcus, Corynebacterium, Neisseria, Actinobacillus*, and *Selenomonas* increased in the upper airway of COVID-19 patients in comparison with healthy participants. On the other hand, *Haemophilus* and *Alloiococcus* abundance decreased in COVID-19 patients with respect to healthy participants. In the lower airway region, the abundance of *Prevotella, Staphylococcus, Haemophilus*, and *Enterococcus* increased, while that of *Veillonella* spp. decreased in fatal infections. During severe but non-fatal conditions, an increased abundance of *Streptococcus, Neisseria, Abiotrophia*, and *Actinobacillus* was observed. The differential bacterial abundance in the nasopharyngeal cavity was also shown during severe SARS-CoV-2 infection (ICU patients) with respect to mild COVID-19, other coronavirus infections, and uninfected individuals (Rueca et al. 2021). Furthermore, *Fusobacterium periodonticum* population depletion, resulting in the perturbation of sialic acid metabolism, has been observed in the nasopharyngeal region of COVID-19 patients with respect to healthy people (Nardelli et al. 2021, Gupta et al. 2022). Another recent study also indicated this pattern, where bacterial species such as *Corynebacterium accolens* and *C. macginleyi* gradually decreased in the upper airway region as the severity of SARS-CoV-2 infection increased (Shilts et al. 2022). An increase in opportunistic pathogens during SARS-CoV-2 infection has been shown to include fungal co-infection (Miao et al. 2021) in addition to the opportunistic bacterial infections mentioned above (Liu et al. 2021). Bronchoalveolar fluid lavage and endotracheal aspiration analysis showed infection of these spaces by *Candida* spp., *Aspergillus* spp., and *Cryptococcus* spp., among all patients (Miao et al. 2021). In severe cases leading to the death of patients, these fungi appeared to co-infect the circulatory systems as well, causing candiduria and candidemia. Miao et al. (2021) also showed that prolonged intubation and mechanical ventilation, rather than SARS-CoV-2 infection, resulted in a less diverse microbiome but a greater enrichment of non-fermentative bacteria such as *Acinetobacter* spp., *Pelomonas* spp., *Ralstonia* spp., and *Sphingomonas* spp. The study indicated the importance of shorter mechanical ventilation to maintain a normal airway microbiome, and likely prevent co-infections in patients infected with SARS-CoV-2.

When multi-omic analyses were applied to such conditions, it was observed that opportunistic pathogens such as *Prevotella histicola, Streptococcus sanguinis*, and *Veillonella dispar* increased in the nasopharyngeal region during COVID-19 infections. In addition, the decreased abundance of natural commensals such as *Gemella haemolysans* and *Leptotrichia hofstadii* was found to be correlated with serum chlorogenic acid methyl ester (CME) depletion (Liu et al. 2021). Our study utilizing ferret models has indicated that the citric acid cycle, purine metabolism, and pentose phosphate pathways were significantly impacted in the nasal cavity during SARS-CoV-2 infection. On the other hand, glutamate and arginine metabolism increased during the infection (Beale et al. 2021), indicating the likely hijacking of glutamate metabolism by viral particles (Thaker et al. 2019), and the promotion of pathogens such as *Pasteurella multocida* (Ren et al. 2013). Overall, these studies indicated that alterations in the lung microbiome can be used as biomarkers of the severity of SARS-CoV-2 infection (Hernández-Terán et al. 2021).

Gut-lung axis although largely remained a neglected area of research, has gained considerable ground since the advent of COVID-19. The SARS-CoV-2 viral has been reported by numerous studies to be found in stool samples (Jin et al. 2020, Pan et al. 2020), with some patients even showing digestive issues post-infection (Jin et al. 2020). Metagenomic analysis of faecal samples collected from COVID-19 patients revealed elevated levels of *Coprobacillus* spp., *Clostridium ramosum, C. hathewayi, Actinomyces viscous*, and *Bacteroides nordii* which were associated with the severity of COVID-19 symptoms (Walton et al. 2021). Additionally, hepatic injury also has been shown in COVID-19 patients (Groff et al. 2021). Altered angiotensin-converting enzyme 2 (ACE2) receptor expression, which has been seen in lung injury during COVID-19, has also been observed in the gut, across numerous studies (Jin et al. 2021), leading to leaky gut and microbiome dysbiosis (Penninger et al. 2021), among other disorders.

Although COVID-19 infections in the lungs and gut occur independently of each other, the underlining cross-linkage still exists, as shown by non-COVID-19 studies. Functional gastrointestinal disorders (FGID) studies have shown that genes related to asthma, such as DENND1B, SMAD3, SLC22A4/5 (5q31/IBD5), and ORMDL3, have been co-associated with Crohn's disease and ulcerative colitis (Lees et al. 2011). This co-occurrence has been ascribed to the common embryonic origin of cellular structures in these organs. Both bronchus- and gut-associated lymphoid tissues (BALT and GALT) are subtypes of mucosa-associated lymphoid tissue (MALT) (Keely et al. 2012, Papanikolaou et al. 2014). This relationship has been observed even more starkly in patients who have undergone colectomy (Papanikolaou et al. 2014), and high intestinal permeability (Adenis et al. 1992).

In the context of infections, the crosstalk of the gut-lung axis is even more explicit. In the study conducted by Sencio et al. (2020), it was seen that the faecal transplantation done from Influenza infected mice to healthy mice caused significant gut microbiome dysbiosis. In turn, it caused an SCFA depletion in the gut, followed by an SCFA depletion in the blood and lungs. The cascade not only depleted alveolar macrophage activity but also increased host susceptibility toward pneumococcal infection. Furthermore, when acetate was supplemented in the gut, it improved the alveolar macrophage activity (Sencio et al. 2020). Similarly, supplementation of desaminotyrosine, a metabolite generally produced by gut *Clostridium* spp. in an influenza mouse model, has been shown to diffuse to the lungs through circulation. The metabolite when diffused into the lungs increased type I IFN–stimulated genes (ISGs). Resultingly, the mortality rate due to influenza infection was seen to be significantly decreased in the mice fed with desaminotyrosine for 1 week before infection, when compared to antibiotics-treated mice (Steed et al. 2017). In the context of COVID-19 infections, there is a clear crosstalk between the gut and lungs. Recent studies have shown that COVID-19 patients, even after recovery, exhibit depleted populations of butyrate-producing bacteria in the gut, such as *Faecalibacterium* spp., *Clostridium* spp., and *Eubacterium* spp. (Tang et al. 2020) compared to non-COVID-19-infected individuals (Zuo et al. 2021). This correlation was also shown by Gutiérrez-Castrellón et al. (2021) who found that prebiotic supplementation with *Lactiplantibacillus plantarum* and *Pediococcus acidilactici* strains not only reduced diarrhea and abdominal pain, but also decreased viral loads in the lower nasopharyngeal cavity, and high SARS-CoV2-binding IgG and IgM titres in the serum. Conversely, COVID-19 patients have also been observed to experience gut dysbiosis that is reflected in their lung health. In a study by El Moheb et al. (2020), COVID-19 patients with acute respiratory distress syndrome (ARDS) developed twice as much transaminitis and severe ileus, and slightly higher bowel ischemia/mesenteric ischemia compared to non-COVID-19 ARDS patients (El Moheb et al. 2020). This is likely due to the high accumulation of ACE2 receptors in gut enterocytes, making the gut an important target for SARS-CoV-2 infection and replication (Zhang et al. 2020).

Given the crucial role of gut microbiota in modulating host immunity and susceptibility to respiratory infections, there is a growing interest in identifying strategies to maintain a healthy and diverse microbiome. Probiotics and prebiotics, for instance, have been proposed as potential interventions that can promote beneficial bacterial growth and improve gut barrier function. Similarly, Ayurveda, an ancient Indian medical system, emphasizes the importance of diet and lifestyle modifications to maintain a balance of doṣas, which has been linked to overall health and disease prevention. In the following sections, we will discuss the current evidence on the potential of these interventions in modulating the gut-lung axis and improving respiratory health.

## Role of probiotics and prebiotics on the gut microbiome

Probiotics and prebiotics have gained greater attention to maintain a healthy and diverse microbial composition in recent years. They have been developed as a preventive approach to maintaining health and well-being based on growing evidence that the gut microbiome plays a significant role in homeostasis (Zucko et al. 2020).

### Probiotics: definition, properties, and mechanism of action

According to the Food and Agriculture Organization (FAO) and the World Health Organization (WHO), probiotics are ‘live microorganisms that confer health benefits to the host when administrated in adequate amounts’ (Sanders 2008, Kechagia et al. 2013). *Lactobacilli, Bifidobacteria*, and other lactic acid-producing bacteria (LAB) have been traditionally used as probiotics and have been primarily isolated from fermented dairy products and the faecal microbiome. To confer the beneficial effects of probiotics, a bacterial strain should (a) demonstrate antimicrobial activity against pathogenic bacteria, (b) be acid- and bile-tolerant, (c) adhere to mucosal and epithelial surfaces for immune-modulatory action, and (d) possess bile salt hydrolase activity (Kechagia et al. 2013, George Kerry et al. 2018).

The mechanism of action of probiotics is quite complex and interactions occur with the host as well as the microbiome via the molecular effectors present on the cell structure or secreted as metabolic products (Aureli et al. 2011, Cunningham et al. 2021). Probiotic molecular effectors influence the microbiota by enhancing the intestinal barrier integrity, modulating the inflammatory signalling system, inducing the production of cytoprotective heat shock proteins, and regulating apoptosis.

Probiotic metabolites can influence the microbiota by changing the micro-environment of the gastro-intestinal tract, competing for nutrients, and binding sites, inhibiting growth via the production of antibacterial compounds, such as bacteriocins, and maintaining the vaginal and oral mucosa microbiomes where pathogen overgrowth is common.

### Probiotics in respiratory tract infections

To understand the mechanism of action of probiotics on respiratory tract infections (RTIs), it is important to note that they operate through the gut-lung axis. Although the exact mechanism is not yet fully understood, it is known that probiotics modulate mucosal immune function, particularly by influencing dendritic cell polarization in inductive sites such as Peyer's patches and mesenteric lymph nodes. These dendritic cells, in turn, influence T and B cell responses. Once these T and B cells enter circulation, they migrate to extra-intestinal sites such as the respiratory tract. Various studies have shown that probiotics can effectively reduce virus titer in the lungs and inhibit the replication of several respiratory viruses, including influenza viruses and respiratory syncytial virus (RSV) (Lehtoranta et al. 2020, Trottein and Sokol 2020, Darbandi et al. 2021, Picó-Monllor et al. 2021). Probiotics such as *Lactobacillus rhamnosus* GG, *L. rhamnosus* Lc705, *Bifidobacterium breve* 99, *B. lactis* BB-12, *B. lactis* Bl-04, *Levilactobacillus brevis* KB290, *L. acidophilus* and *Propionibacterium jensenii* JS have shown a potential reduction in rhinovirus-induced illness (Lehtoranta et al. 2020). A similar response for SARS-CoV-2 was associated with prebiotics such as *Lachnospiraceae bacterium, Eubacterium rectale, Ruminococcus obeum, Alistipes onderdonkii, Leuconostoc spp*., *Pediococcus pantosaceus, Enterococcus spp*. and *Dorea formicigenerans* (Trottein and Sokol 2020).

As stated previously, ACE2 expression is down-regulated in patients during SARS-CoV-2 infection. Downregulation of ACE2 impairs the conversion of angiotensin II to angiotensin(1–7), which further inhibits the angiotensin II receptor type 2 (AT2R) that provides lung protection. This stimulates angiotensin II receptor type 1 (AT1R) which further leads to hypokalemia, causing lung and cardiovascular injury (Silhol et al. 2020). ACE2 is also found in the epithelial cells of the gut and regulates the expression of amino acid transporters that control the intestinal uptake of tryptophan. Tryptophan further regulates anti-microbial peptides; down-regulation leads to impairment in the absorption of intestinal tryptophan and decreases secretion of antimicrobial peptides and, thus, increases the survival of pathogenic microorganisms and causes dysbiosis (He et al. 2020, Zhou et al. 2021). However, the exact mechanism of probiotics is not yet established in RTIs, however, the effects are influenced by specific strains, microbiota composition, and immune status of an individual (Lehtoranta et al. 2020).

### Prebiotics

Prebiotics are defined as non-digestible foods such as plant oligosaccharides that pass intact into the intestinal tract and beneficially affect the host by selectively stimulating the growth, composition, and activity of intestinal microbiota, improving host health (Peredo-Lovillo et al. 2020). Plant oligosaccharides and polysaccharides are fermentable and indigestible fibers that feed the intestinal microbes and contribute to SCFA production and thus are good sources of prebiotics. Examples of foods used as prebiotics are given in Table 1 (Batista et al. 2021, Pujari and Banerjee 2021, Walton et al. 2021).

**Table 1.** Plant constituents with examples of food as prebiotics

Plant Constituents	Foods
Inulin	Asparagus, onion, wheat, tomatoes, garlic, barley, and chicory roots
Fructo-oligosaccharides (FOS)	Sugarcane, beetroot, asparagus, Jerusalem artichoke, rye, garlic, onions, chicory, wheat, and banana
Galacto-oligosaccharides (GOS)	Human milk, and cow milk
Beta-glucans	Cereals, grains, mushrooms, algae, yeast, and other marine plants
Isomalto-oligosaccharides	Sugarcane, honey, and starch
Xylo-oligosaccharides	Vegetables, bamboo shoots, fruits, wheat bran, and honey
Arabinoxylo-oligosaccharides	Wheat bran
Resistant starch	Raw potatoes, green bananas, and grains

Fermented food products (e.g. butter, cheese, yogurt, milk, lentils, meat, fish, and sourdough bread) have been consumed since pre-historic times. The benefits of fermented foods have been mentioned in ancient texts such as Āyurveda, the Bible, and archaic text from Uruk (Lemmen and Khan 2012). Fermented foods (e.g. sauerkraut, kimchi, tofu, ghee and tempeh) and beverages (e.g. kefir—fermented milk, kaanji—fermented carrot drink with spices, kombucha—fermented tea, toddy—fermented palm nectar, koji—fermented drink of sweet potato, rice, or barley, merissa—fermented product of malted sorghum and jiu—fermented drink of sorghum) have been used traditionally (Gasmi et al. 2021, Ilango and Antony 2021, Pammi et al. 2021). Various beneficial microorganisms such as lactic acid bacteria, yeast, and *Rhizopus* spp. have been isolated from fermented foods (Ilango and Antony 2021).

The understanding of prebiotics has constantly been updated and shows that the effects are indirect and regular consumption of prebiotics can be used as a potential strategy for maintaining general health and preventing diseases (Cheng et al. 2022). Metabolism of fermentable fibers produces SCFAs including butyrate, propionate, and acetate that regulate the intestinal barrier, produce anti-inflammatory signalling molecules, and improve the bioavailability of vitamins and minerals. Cellulose and lignins also increase the bulk of faecal matter and increase the transit time (Lama Tamang et al. 2022).

Several dietary substances have the potential to act as prebiotics and must confer the following properties: must be fermented and not absorbed in the upper part of the gastrointestinal tract; must selectively stimulate the growth of beneficial bacteria; must change the intestinal microbiota of the colon to make it healthier; must induce systemic effects beneficial to the health of the host (Cheng et al. 2022). Food processing, pH, amount of salt, sugar, food additives, preservatives, temperature, moisture, packaging, and water content of the food are all factors that can affect prebiotic properties (Ugural and Akyol 2022).

Polyphenols, such as anthocyanins, flavonoids, and flavanones, act as prebiotics by favouring the growth of beneficial bacteria and regulating the diversity of intestinal bacteria. Epigallocatechin (EGCG) showed anti-viral activity including inhibition of SARS-COV-2 (Xu et al. 2022). EGCG (a compound found in green tea), green tea extracts, and Kimchi (Korean fermented food) have been found to have potential benefits in suppressing SARS-CoV-2 (Jaffal and Abbas 2021). This is achieved by inhibiting the main protease (M^Pro^, also known as 3C-like protease), which is crucial for the virus's life cycle. Additionally, these substances provide mitochondrial protection by suppressing pro-oxidant enzymes, activate the cytoprotective transcription factor pathway known as nuclear factor erythroid 2 p45-related factor 2 (NrF2) pathway and downregulate ACE2 and transmembrane serine protease 2 (TMPRSS2). By reducing the oxidative stress and cytokine storm, they can lower the risk of pulmonary fibrosis, thrombosis and sepsis in COVID-19 patients (Zhang et al. 2021). Fermented vegetables and Brassica family vegetables contain sulforaphane that activates the NrF2 pathway and, hence, has shown viral protection and protection against early oxidative stress and thus can help mitigate the severity of COVID-19 (Kesika et al. 2022), though more evidence is needed towards the diet as a preventive approach for COVID-19 patients.

### Synbiotics

Synbiotics are combinations of live microorganisms added to fermentable foods, which can have synergistic effects on health. There is a growing interest in the food industry to develop products that can improve gut health. Mixtures of living or dead microorganisms with fermentable substrates, vitamins, minerals, polyphenols, or other phytochemicals can provide dietary supplements that can help manage the delicate balance between health and disease (Cunningham et al. 2021). Synbiotics represent a novel strategy to promote the growth of healthy bacteria through prebiotics while simultaneously enriching the growth and colony of healthy bacteria through probiotics.

A synbiotic formulation consisting of *L. plantarum, L. acidophilus, L. reutri*, and prebiotic fibers inulin and fructo-oligosaccharides (FOS) for 2 months has been shown to deplete the markers of insulin resistance related to metabolic syndrome and several cardiovascular risk factors (Cunningham et al. 2021). To further investigate the potential of microbiome regulation as a treatment strategy for COVID-19, a clinical study was conducted utilizing a novel symbiotic formula (SIM01) (Zhang et al. 2022). This formula contained *Bifidobacterium* strains, galactooligosaccharides, xylooligosaccharides, and resistant dextrin, and was administered to hospitalized COVID-19 patients. Results showed a significant reduction in inflammatory markers, an improvement in antibody formation against SARS-CoV-2, and a reduction in nasopharyngeal viral load (Zhang et al. 2022). Additionally, the symbiotic formula was effective in restoring gut dysbiosis in these patients, indicating the potential for microbiome-targeted therapies in the treatment of COVID-19 (Zhang et al. 2022). Therefore, the innovation and development of functional foods such as synbiotics can provide a beneficial approach to health promotion and wellness amongst patients with RTIs.

## Role of ancient Indian medicinal knowledge on the microbiome

### *‘We are what we eat & drink’:* the ayurvedic concept of gut health

We now recognize that virtually every aspect of our physiology and health is influenced by the collection of microorganisms that live in various parts of our body, especially the gut microbiome. Of numerous external factors influencing the gut microbiome composition, diet and digestion are perhaps the most important. Although Āyurveda does not mention the gut microbiome explicitly, nevertheless, this traditional system of medicine, which literally translates to ‘*knowledge of life*’, has focused on diet and digestion for thousands of years.

Āyurveda highly values the right food, digestion, and all other facets of lifestyle which are bound to affect gut health. Interestingly, some modern scientists have suggested that the practice of Āyurveda is a form of ancient epigenetics (Sharma 2016). Although the Āyurvedic practitioners may not have been aware of the precise molecular mechanisms by which food could have affected gene expression, nevertheless, they did understand that each individual has a unique psychophysiological constitution that is influenced by factors such as diet, digestion, lifestyle, stress management, and environmental factors (Bordoni and Gabbianelli 2019). Modern research is helping us to understand the relationship between the microbiome and various preventions and treatment approaches of Āyurveda (Wallace 2020).

Tridoṣa theory in Āyurveda is the foundation for explaining the functioning of the human mind and body (Dash and Sharma 1995). The theory posits that three fundamental principles of physiology, Vāta, Pitta, and Kapha, govern our body and mind, and are identified as *doṣas*. The theory explains how these *doṣas* interact and influence disease manifestation and progression, with a focus on the host rather than the illness (Fiandaca et al. 2017).

Each of these *doṣas* is associated with physiology-specific attributes. The distribution profile of the three *doṣas* at birth, with one *doṣa* more dominant than others, is known as *Prakriti* in Āyurveda and is one of the first steps in evaluating a person's health. Any deviation from *Prakriti* or natal *doṣa* distribution profile is recognized as *Vikriti*. The *Prakriti* assignment entails phenotyping a person based on their physical type, dietary and bowel habits, ability to fight off sickness, healing, memory capacity, metabolism, and other traits (Dey and Pahwa 2014). Thus, finding distinctive *Prakriti*-specific microbial fingerprints is an intriguing foundation for customized therapy (Jnana et al. 2020).

These methods of Āyurvedic therapy are comparable to current treatment trends in modern medicine, which emphasize food and lifestyle modifications as a means of reducing sickness (David et al. 2014, Conlon and Bird 2015, Shondelmyer et al. 2018). With advanced ‘omic’ platforms, modern medicine has the scope of improving customized treatments based on the genetic and epigenetic profiles of the patients. The Tridoṣa theory would possibly be the Āyurveda equivalent of *precision medicine*, wherein Ayurvedic practitioners/physicians recommend individualized therapy based on a patient's *Prakriti* (Rotti et al. 2014). Converging evidence associates specific chromosomal (Govindaraj et al. 2015), epigenetic (Rotti et al. 2015), and biochemical (Prasher et al. 2008) properties with specific *Prakriti* profiles, supporting the idea of customized care in the Āyurvedic system. It's important to note that Āyurveda considers each individual to be unique, therefore, the approach to health and wellness is highly personalized. While Āyurveda doesn't directly link specific diseases to specific *Prakriti* types, it suggests that certain imbalances may be more likely in individuals with certain constitutions. The gene expression profiles vary significantly between the three *doṣas*.

Studies have shown that each Vāta, Pitta, and Kapha primary *Prakriti* type has a distinct microbiome composition (Chauhan et al. 2018, Chaudhari et al. 2019, Shalini et al. 2021). Prior research has demonstrated the impact of environment and dietary behaviours on the composition of the gut microbiome (Senghor et al. 2018). The maintenance of homeostasis, digestion and metabolism, detoxification, and many other biological processes are all greatly influenced by the gut microbiome, which has been proven to have a complex biochemical interaction with its host (Nicholson et al. 2012). Thus, variations in *Prakriti* are very likely to affect gut microbiome dysbiosis. Therefore, *Prakriti* phenotyping, as done in Āyurveda by precisely associating different *Prakriti* types with phenotypic attributes, could be an effective strategy to predict the stratification of the gut microbiota in a specific population (Jnana et al. 2020). In a modern context, these phenotypic attributes identified by Āyurveda for each *Prakriti* type have been validated through metagenomics studies (Chauhan et al. 2018, Mobeen et al. 2019). The *Prakriti* phenotype-based therapy of Āyurveda is founded on the idea of a *Prakriti*-specific microbiome, and treatment is provided considering the patient's gender and overall health, among other things. *Prakriti*-specific microbiome often serves as nature's prophylactic measure towards disease pathogenesis.

### ‘Let food be thy medicine and medicine be thy food.’ ― hippocrates

Since the gut is believed to be the origin of all illnesses, food is regarded as medicine in Āyurveda. Healthy eating is an integral part of Āyurvedic therapies. Besides a plethora of medicinal herbs, Āyurveda employs a variety of spices, herbs, mineral salts, and other natural products, which are often everyday food items in themselves, to aid in restoring and maintaining physiological equilibrium and treating certain illnesses.

Āyurvedic medicines are mostly polyherbal or herbs-mineral formulations (Agnivesha 2001, Dr. P. Himasagarachandramurthy 2001, Ayurvedic Formulary of India 2003, Satyapal B 2004, Pt. Krishna Gopalacharya 2005, Shastri 2006, Samhita 2009, Tiwari 2013, Gaur et al. 2014, Sharma 2020). There are numerous formulations (Table 2) known for their effectiveness against gut disorders such as irritable bowel syndrome (IBS) (Tiwari et al. 2013, Das et al. 2019) and respiratory disorders such as asthma (Erickson and Jarvis 2019), with early studies showing inhibition of SARS-CoV-2 main protease (M^Pro^) (Ram et al. 2022). Overall, a combination of herbs and other natural ingredients, together with the process of preparation makes these formulations different from each other, and selectively effective in a *Prakriti*-specific treatment.

**Table 2.** Different Ayurvedic formulations for gut health

Ayurvedic Formulations	Composition	Natural Ingredients	Disease / Therapeutic Area	Reference
Marichyadi Churna	Polyherbal	*Piper nigrum (black pepper), Zingiber officinale (ginger), and Holarrhena antidysentrica*	Irritable bowel syndrome	(Wang et al. 2016)
Takrarista	Polyherbal	*E. officinalis (Indian gooseberry), Trachyspermum ammi (Carom), Terminalia chebula (Chebulic Myrobalan) and Piper nigrum (black pepper)*	Irritable bowel syndrome	(Whipps et al. 1988)
Pippalyasava	Polyherbal	*Piper longum (long pepper), Piper nigrum (black pepper), Curcuma longa (turmeric), Piper chubeba (cubeb pepper), Plumbago zeylanica (doctorbush), Symplocos racemose (Symplocos), Cyclea peltata (Indian moon-seed), Cyperus rotundus (nut grass), Embelia ribes (false black pepper), Areca catechu (betel nut), Emblica officinalis (Indian gooseberry), Elettaria cardamomum (cardamom), Vetiveria zizanioides (Vetivergrass), Santalum album (sandalwood), Saussurea lappa (Indian Costus tree), Syzygium aromaticum (clove), Valeriana wallichii (Indian Valerian), Cinnamomum zeylanicum (cinnamon), Mesua ferrea (Indian Rose Chestnut) and Callicarpa macrophylla (perfumed cherry)*	Digestive problems	(Winter et al. 2013)
Abhayarishta	Polyherbal	*Terminalia chebula (chebulic myrobalan), Vitis vinifera (grape), Embelia ribes (false black pepper), Madhuca indica (honey tree), Piper retrofractum (Javanese long pepper), Operculina turpethum (Transparent Wood Rose), Baliospermum montanum (wild castor), Woodfordia fruticosa (Fire-flame bush), Tribulus terrestris (puncture vine), Citrullus colocynthis (bitter apple), Salmalia malabarica (Red Silk cotton tree), Coriandrum sativum (coriander), Foeniculum vulgare (fennel) and Zingiber officinale (ginger)*	Digestive problems	(Wu et al. 2011)
Dadimashtaka Churna	Polyherbal	*Punica granatum (pomegranate), Cinnamomum zeylanicum (cinnamon), Trachyspermum ammi (Yavani), Coriandrum sativum (coriander), Piper nigrum (black pepper), Piper longum (long pepper), Zingiber officinale (Ginger), Bambusa bambos (Indian thorny bamboo), Mesua ferrea (Indian Rose Chestnut), Cuminum cyminum (cumin), Cinnamomum tamala (Indian bay leaf) and Elettaria cardamomum (cardamom)*	Diarrhea	(Wypych et al. 2019)
Bhunimbadi Churna	Polyherbal	*Swertia chirayita (East Indian Balmony), Holarrhena pubescens (Ester Tree), Zingiber officinale (Ginger), Piper nigrum (black pepper), Piper* 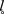 *ongum (long pepper), Cyperus rotundus (nut grass), Picrorhiza kurrooa (Hellebore), Plumbago zeylanica (doctorbush) and Wrightia antidysenterica (Arctic Snow)*	Diarrhea, malabsorption syndrome, Jaundice, low appetite, dysentery, and loose motion with blood	(Xu et al. 2022)
Chitrakadi vati	Herbo-mineral	*Herbs:* *Plumbago zeylanica (Doctorbrush), Apium graveolens (Celery), P. nigrum (Black pepper), Piper chaba (Java Long Pepper), Piper longum (Long pepper), Zingiber officinalis (Ginger), and Ferula asafetida (Asafoetida), ‘Yavkshaar’, an alkaline preparation made from whole plant of barley (Hordeum vulgare) and lemon (Citrus limon) or pomegranate (Punica granatum) juice* *Minerals/Salts:* *Earthen salt, Common salt, Black salt, Rock salt, Black salt with traces of Sodium sulphate, Sodium bicarbonate*	Indigestion, constipation, anorexia, abdominal distension, stomatitis, stomach discomfort	(Yagi et al. 2021)
Swadishta Virechana Churna	Herbo-mineral	*Herbs:* *Glycyrrhiza glabra (Liquorice), Foeniculum vulgare (Fennel), Cassia angustifolia (Indian Senna)* *Minerals/Salts:* *Purified Sulphur and sugar*	Purifies the toxic load built up in the body	(Yatsunenko et al. 2012)
Avipattikar Churna	Herbo-mineral	*Herbs:* *Emblica officinalis (Indian gooseberry), Terminalia bellirica (Beleric myrobalan), Piper nigrum (black pepper), Elettaria cardamomum (cardamom), Syzygium aromaticum (clove), Zingiber officinale (ginger), Terminalia chebula (Chebulic Myrobalan), Cinnamomum tamala (Indian bay leaf), Piper* 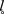 *ongum (long pepper), Cyperus rotundus (nut grass), Operculina turpethum (Transparent Wood Rose), Embelia ribes (false black pepper)Minerals/Salts:Crystallized rock sugar or mishri*	Carminative, antioxidant, and anti-inflammatory; treat ulcers, gastritis, indigestion, heartburn, diarrhoea, and constipation	(Yip et al. 2021)
Lavan Bhaskar Churna	Herbo-mineral	*Herbs:* *C. sativum (coriander), P*. 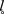 *ongum (Long pepper), Bunium persicum (Caraway), C. tamala (Indian bay leaf), M. ferrea (Indian Rose Chestnut), Abies spectabilis (Himalayan silver fir), Garcinia pedunculata (gamboge), C. cyminum (cumin), Z. officinalis (Ginger), P. granatum (pomegranate), C. zeylanicum (cinnamon), E. cardamomum (cardamom), Minerals/Salts:Black salt, sea salt, rock salt and salt isolated from alkaline soil*.	Gastritis	(Yoshimoto et al. 2013)

### Rituchariya: integrating season, diet, and lifestyle to maintain a healthy gut microbiome

According to Āyurveda, *Agni* (digestive fire) and *Āma* (metabolic toxins) are central to an individual's health. Āyurveda states that all illnesses are the results of dysregulated *Agni*. Thus, *Agni* modulation is central to both preventative and treatments in Āyurveda. *Āma* accumulation is a result of out-of-sync *Agni*, and their dissipation throughout the body causes detrimental effects and a myriad of ailments. In the context of modern science, this concept is equivalent to gut dysbiosis leading to a leaky gut, allowing the metabolic toxins of dysregulated digestion to enter systemic circulation, and manifest as ailments. Metabolic toxin accumulation could be a result of unhealthy food habits, lifestyle, or environmental factors such as seasonal changes.

Āyurveda recommends preventive medication based on the concept of Shatkriyākalā, dynamic prophylaxis co-ordinated to stages of disease prognosis (Shata = six, Kriyā = choice of treatment, Kalā = stage of progress of disease). Six stages of progression during a disease that is represented in modern science as oxidative stress, alteration in immune functions, and changes in anatomical and physiological mechanisms that further lead to the manifestation of a disease and progression of complications. Shatkriyākalā offers the opportunity to tackle disease progression at every step with adequate and strategized intervention through integrated treatment approaches that take into consideration the season, diet, lifestyle, and medication. The Ayurvedic practice of modulating diet and lifestyle according to the season is called Ritucharya (pronounced as 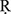
tucaryā), the seasonal lifestyle programme. Ritucharya can effectively regulate the gut microbiome to keep an individual healthy in all seasons. The Āyurvedic concept of eating with the seasons is crucial, as it emphasises that various foods are beneficial at different times of the year (Deepthi Rani et al. 2021). Eating seasonal and locally produced foods can support the body's natural rhythms, promote overall health and well-being, and reduce the environmental impact of food production(Macdiarmid 2014, Deepthi et al. 2021). Moreover, importing non-seasonal food may contribute to imbalances in the body and potentially lead to dysbiosis (Macdiarmid 2014). The recent review by Wallace (2020) is one of the earliest modern science-based reports to indicate the importance of Ritucharya in COVID-19′s context, particularly during a post-infection recovery regimen. The recent network pharmacology study reported by Khanal et al. (2022) has also indicated the impact of Ritucharya-based food intake on the modulation of HIF-1, p53, PI3K-Akt, MAPK, cAMP, Ras, Wnt, NF-kappa B, IL-17, TNF, and cGMP-PKG signaling pathways, to improve the recovery from COVID-19 infection.

Altogether, it is getting increasingly evident that lifestyle and dietary habits significantly change the commensal microbial populations of the human gut, and a dysbiosis of these communities can increase pathogen susceptibility, inflammatory disorders, and the current pandemic of metabolic health problems, including non-communicable diseases. Adopting Ritucharya, which integrates lifestyle and diet with seasonal variations, would allow the host to build a season and *Prakriti*-specific gut microbial profile as a shield against diseases and infections.

## Conclusions

The human gut microbiome is a dynamic entity; whose compositional equilibrium requires constant buffering from extrinsic and intrinsic modulators. A breach in this equilibrium can snowball into health challenges, as evidenced during the COVID-19 pandemic. The advancement in technologies has undoubtedly empowered us in precisely identifying the alteration in the homeostatic profile. Nevertheless, restoring a dysbiotic gut microbiome is still an unmet medical need at several levels of our contemporary scientific understanding. However, an alternative approach of delving into the traditional knowledge of healthy living could provide a mechanism to balance the ‘*imbalance*’ predicament. Āyurveda, the traditional Indian system of medicine, and its theories and principles regarding gut health maintenance have indeed enlightened the potential strategies for addressing gut-lung axis dysbiosis. The practice of co-existing with nature and the time-honored principles of Āyurveda can provide a solid foundation for maintaining a healthy gut microbiome. By returning to these roots, we may find the key to balancing our gut microbiome and achieving optimal health.
